# Public Awareness about Medicine Information, Safety, and Adverse Drug Reaction (ADR) Reporting in Dammam, Saudi Arabia

**DOI:** 10.3390/pharmacy8040222

**Published:** 2020-11-18

**Authors:** Md. Ashraful Islam, Aseel Fuad Al-Karasneh, Atta Abbas Naqvi, Dhfer Mahdi AlShayban, Fatimah Al-Hayek, Sarah Al-Badrani, Raghad Al-Salem, Syed Azizullah Ghori

**Affiliations:** 1 Department of Pharmacy Practice, College of Clinical Pharmacy, Imam Abdulrahman Bin Faisal University, Dammam 31441, Saudi Arabia; afalkarasneh@iau.edu.sa (A.F.A.-K.); naqviattaabbas@gmail.com (A.A.N.); dmalshayban@iau.edu.sa (D.M.A.); agsayed@iau.edu.sa (S.A.G.); 2 College of Clinical Pharmacy, Imam Abdulrahman Bin Faisal University, Dammam 31441, Saudi Arabia; hayekf97@gmail.com (F.A.-H.); sarah.badrani@gmail.com (S.A.-B.); raghadali922@gmail.com (R.A.-S.)

**Keywords:** pharmacovigilance, adverse drug reporting, medicine information, Saudi Arabia

## Abstract

This study aimed to assess public knowledge about medicine information, safety, and adverse drug reaction reporting (ADR) in Dammam, Saudi Arabia. A cross sectional study was conducted using purposive stratified sampling in different settings of Dammam city for three months (January–March 2020). The target population was identified as consumers who had used the medicines in the last 3 months. The questionnaire was adopted from the literature and was validated. Content and face validities were established, and reliability was assessed. The study was approved by the concerned ethics committee. A total of 915 participants returned completed questionnaires. A total of 54.4% participants aged between 18 and 30 years, 65.8% were females and 53.1% had obtained bachelor level education. The mean score for knowledge of medicines (K1) was 5.46 ± 1.07. The mean score for knowledge regarding medication safety (K2) was 5.94 ± 1.73. The mean score for tendency to report a suspected ADR (T1) was 3.43 ± 1.57. Gender was a determinant of knowledge regarding medication safety (K2) (*p* < 0.01) and ADR reporting tendency (T1) (*p* < 0.01). The marital status of patients was a determinant for both knowledge of medicines (K1) (*p* < 0.01) and, knowledge regarding medication safety (K2) (*p* < 0.01). The results of this study highlighted that although the scores for knowledge of medicines, and tendency to report ADR were better, the score for knowledge regarding medication safety was unsatisfactory.

## 1. Introduction

One of the most important aspects of post marketing surveillance is the detection of adverse drug reactions (ADR) through spontaneous reporting (SR) [1]. Available evidence mentions the importance of patient reports as a credible source of information about the safety of new drugs [1]. Spontaneous reporting (SR) is a novel mechanism within the pharmacovigilance system to detect ADRs early; that were not reported previously. These ADRs could be rare and may be life threatening [1,2,3]. Ensuring patient safety is essential in sustaining the business of pharmaceutical industries. Notwithstanding the value of randomized controlled trials (RCTs) in evaluating potential medicines for their therapeutic and safety profile, the design of such studies makes it difficult to monitor ADRs since RCTs are usually conducted in a strictly defined population for a limited time period [1,4,5,6]. Therefore, monitoring for safety after the medicine is approved for use by the regulator is critical to ensure treatment success, as well as minimizing any possible harm [6].

The World Health Organization (WHO) defines the term pharmacovigilance (PV) as, “the science and activities relating to the detection, assessment, understanding and prevention of adverse effects or any other medicine-related problem” [7]. Since 1960, this practice of SR has been followed globally to ensure the continuous monitoring of ADRs [1,7]. One of the major components of SR is data collection which could be voluntary or mandatory, depending upon a country’s regulation [7]. Since 2012, countries in the European Union have involved patients in ADR reporting [1,8]. In the past, healthcare professionals were solely considered to report ADRs. Nowadays, both the WHO and the European Union recognize the importance of direct patient reports [1,2,7].

The Saudi National Pharmacovigilance Center (SNPC) or NPC, was inaugurated in March 2009 in Riyadh, Saudi Arabia [9,10]. Later, the Saudi Food and Drug Authority (SFDA) became a full member in the Uppsala monitoring center (UMC) of the WHO [10,11]. The system is accessible to healthcare providers, pharmacies, health institutions and the Saudi public. Data highlighted that between 2010 and 2017, there was an increase in the number of reports through the system. However, despite these numbers, under-reporting is an issue in the Saudi healthcare sector [11].

Some notable reasons for under-reporting of ADRs include low awareness regarding the reporting system, lack of training, reliance on other health practitioners for reporting and, inability to identify the ADRs [12,13,14,15]. Evidence highlights that patients use the information about medication provided from different resources such as healthcare professionals, online sources, and patient information leaflet (PIL) to help identify a possible ADR [16].

It is unclear how well consumers are aware of the medicines and their safety, as well as the role of Saudi NPC. Moreover, the literature regarding consumers’ perception toward ADR reporting in Saudi Arabia is limited. This study aimed to assess the public knowledge about medicine information, safety and adverse drug reaction reporting in Saudi Arabia.

## 2. Materials and Methods

### 2.1. Study Design and Venue

A cross sectional study was conducted using purposive stratified sampling in different settings of Dammam region for three months (January–March 2020).

### 2.2. Target Population and Eligibility Criteria

The target population was identified as consumers. Consumers from different age groups and genders who had used medicines in the last three months were included in the study. Those consumers who were under 18 years old, had not used medicines in the last 3 months and, not willing to participate, were excluded.

### 2.3. Sampling Strategy and Data Collection

Consumers were stratified into three main strata, i.e., students, employed and general public. The general public further included individuals who were un-employed, home makers and retired. Students were targeted in universities, employed consumers were contacted through email derived from social media while the general public was invited by visiting public places at weekends. The data collection started on 27 January 2020 and stopped on 31 March 2020.

### 2.4. Sample Size Calculation

Sample size was calculated using an online calculator [17]. The target population was identified as general population of Dammam region. According to official estimates the region has a population of 0.77 million [18]. This figure was identified as the target population and considering a 5% error and 95% confidence interval, the required sample size was 384.

### 2.5. Questionnaire Development and Validation

A questionnaire was adopted from the available literature and was further developed and validated [19,20]. The final version consisted of close ended questions covering the demographic information, the use of medications, reading and understanding the PIL, and the perception of drug safety. Apart from the demographic section, the questionnaire had 3 sections: (1) knowledge of medicines, (2) knowledge of medication safety and, (3) tendency to report a suspected ADR. The questionnaire was translated in Arabic using a forward–backward translation method by native Arabic speakers whose second language was English. The questionnaire underwent content and face validation as well as piloting.

The content validity in Table 1 was conducted according to the Lawshe’s method [21]. The content validity ratio (CVR) for items 2, 7, 12, 14 and 19, was 0.75. The CVR for all other items was 0.99. According to the criteria, CVR ≥ 0.75 was acceptable for each item [21]. The content validity index (CVI) was 0.93, i.e., acceptable. Furthermore, face validation was conducted via face-to-face review and some grammatical errors were rectified. The questionnaire was handed to 9 participants for piloting. No difficulty was reported in terms of understanding of the items. The pilot data were not included in the analysis.

### 2.6. Scoring of the Questionnaire

The questionnaire documented scores for three aspects of pharmacovigilance namely, knowledge of medicine (K1), knowledge regarding medication safety (K2) and tendency to report a suspected ADR (T1). The scoring criteria are presented in Table 2.

### 2.7. Data Analysis

The data were analyzed using SPSS version 23. Data were expressed as frequencies (*n*) and percentages (%). Where applicable, inferential statistics such as regression analyses were used to demonstrate the relationship between demographic variables and pharmacovigilance characteristics of participants.

### 2.8. Ethics Approval and Consent

Written informed consent was obtained from participants. The ethical approval was obtained from the Institutional Review Board (IRB) of Imam Abdulrahman Bin Faisal University, Dammam, Saudi Arabia, (IRB-2020-05-018).

## 3. Results

A total of 915 responses were received, the majority of the participants were between 18 and 30 years old (54,4%), females (65.8%), all of the participants were educated but with different levels where (53.1%) had a bachelor’s degree, more information about the participant demographics can be found in Table 3.

### 3.1. Medication Information

The mean score for knowledge of medicines (K1) was 5.46 ± 1.07 while the median was 6.0. Most participants (52.8%) had some medications without prescription and almost a quarter (24.9%) used them as a self-recommendation, followed by some who used them on the recommendation of pharmacist (20.4%). The participants were assessed for their knowledge about, indication, frequency, and duration of medication. The majority were aware of these (86.3%, 84.5%, 74%, respectively). More information about the participant medication information can be found in Table 4.

### 3.2. Medication Safety

The mean score for knowledge regarding medication safety (K2) was 5.94 ± 1.73 and the median was 6. Most of them indicated that they always read it (43.1%) or sometimes read it (49.6%), and only a few reported difficulty reading the PIL (7.3%). The majority was interested in reading the whole PIL (52.9%) but others participants specified other parts such as indication (19.7%), side effects (15.2%), dose and instructions (12.2%). The majority of participants knew about ADRs correctly (37.2%) but some participants (36.3%) answered “any effect of the medication”. Almost a quarter (24.3%) answered “expected reaction after taking the normal dose”. Few (2.3%) did not know what an ADR meant (Table 5).

### 3.3. Adverse Drug Reaction (ADR) Reporting Tendency

The mean score for tendency to report a suspected ADR (T1) was 3.43 ± 1.57 and the median was 4.0. Most (58.7%) believed that both consumers and health care professionals (HCPs) should report ADRs. Some (39%) mentioned that it was the responsibility of the HCP only while a small segment (2.3%) believed it was consumers responsibility. Some participants had a previous ADR experience (16%) and those who had suffered, either reported it to HCPs (8.1%) or did not report it to any facility (7.8%). One participant did report it to the Saudi National Pharmacovigilance Center (NPC). Regarding the participants knowledge about the Saudi NPC, the overwhelming majority of people were not familiar with the center’s existence (91%). They also believed that this option should be available to the public (93.1%), and they expressed their interest to know more about the Saudi NPC (90.5%) (Table 6).

Bivariate analysis revealed that the mean score for knowledge of medicine (K1) was higher in patients above 45 years, those who were employed, and married. The variables of gender and education were not significantly associated with K1. The knowledge about medication safety (K2) was higher in married female patients below 45 years. The variable of education was not significantly associated with K2. The tendency to report a suspected ADR (T1) was higher in married female patients. All other demographic variables were non-significant for T1. The details are mentioned in Table 7.

Multivariate analysis revealed that gender was a determinant of knowledge regarding medication safety and male patients were more likely to have lower knowledge. In addition, the marital status of patients was also a determinant for both knowledge of medicines and knowledge regarding medication safety. Married patients were more likely to have better knowledge about medicines and their safety. The variables of age and employment were non-significant (Table 8).

## 4. Discussion

In our observation, most participants were either self-medicating or had their medications advised by a pharmacist. In a study it was observed that most patients in Saudi healthcare settings seek counseling from pharmacists [22]. Moreover, a dedicated drug information service is available in healthcare settings of the Dammam region [23]. The score indicated adequate knowledge about medication use which was in line with the results reported by Salgueiro et al. [20]. The score suggests good knowledge about medications among patients who were above 45, employed and married. Henceforth, this knowledge could result from more frequent use of prescribed medication in older age-groups. Employed participants may have better opportunities to learn about medicines by exchange of information with colleagues and information about medical insurance they are usually entitled to at the workplace. Similarly, being in a marital relationship allows both participants to be exposed to their medical history, apart from the knowledge exchange. Studies have highlighted that a spouse’s influence may lead to their partners improving their health behaviors [24,25]. Therefore, marital support may have some role in improvement of knowledge regarding medicines. Though, this hypothesis may require further investigation.

The majority (43.1%) of participants reported that they always read the PIL which aligns with the findings of several studies from other countries [20,26,27]. A study in Spain mentioned that 61.4% of participants always read the PIL [20]. A study in England reported that 41.9% of respondents always read the PIL [26]. A study in USA reported that 49.2% of participants always read the PIL [27]. Most participants (52.9%) in this study emphasized their interest in reading the whole PIL, however, other studies showed that the section pertaining to the side effects is the most read part of PIL [26,27,28]. A study by Krska and Morecroft reported that 37.7% of participants in England read the PIL section related to side effects [26]. While a study by Nathan and colleagues reported that 60.7% of respondents in the USA read the same section of the PIL [27]. Raynor and colleagues mentioned that 60–66% participants in the UK read the section related to side effects [28]. A study in UK examined PIL for appropriateness of information related to ADR risk presentation and found that few leaflets had this information according to the guidelines [29]. In this study, only 15.2% of participants read the side effects section, while the section that was read by most respondents (19.7%) was the indication of the medicine. Only few reported having difficulty reading and understanding the PIL.

Most participants (37.2%) accurately defined ADRs, however some were confused about the ADR meaning. This finding was in contrast to those reported by Sales et al. in Riyadh where majority defined ADR incorrectly and only a quarter (25.7%) knew the correct definition [19]. The score indicated a satisfactory knowledge of medication safety among participants. Further, it was evident that respondents had an interest in asking about possible ADRs of medicines they used. They showed interest in seeking information from the most common platforms, namely the internet, as well as the PIL, followed by healthcare professionals (HCPs) such as doctors and pharmacists. The majority mentioned that both HCPs and the consumers were responsible of reporting ADRs, though it was observed that a large number of participants rely on HCPs as responsible persons to report potential ADRs. In the study by Sales and colleagues, most respondents (73.2%) mentioned that ADR reporting should be done by HCPs [19].

Previous studies show a low tendency of ADR reporting among HCPs as well as their knowledge about the SNPC [30,31,32]. Therefore, it is imperative that both HCPs and consumers be educated about the process of reporting of ADRs. Mahmoud and colleagues mentioned the need to formulate interventions to improve knowledge of ADR reporting [32]. The study reported that less than 10% of participants were aware of the SNPC which was even lower than the figure reported by sales et al., i.e., 15.1% [19]. Alharf et al. mentioned that awareness regarding NPC could be created among HCPs through workshops. Moreover, establishment of regional committees to facilitate a knowledge exchange between NPC and HCPs would enhance communication [10]. The results of this study have policy implications as they have highlighted that the public has a low awareness about the NPC. Alharf et al. mentioned that social media could be helpful in disseminating information among the masses [10]. Therefore, various social media platforms could be used to provide awareness. In addition, communicating with HCPs to educate patients about the NPC and ADR reporting process during counseling could be very helpful in increasing ADR reporting. The results of this study highlighted that females had a greater tendency to report ADRs. This notion directs the attention to the male members of the public and targeting this stratum with the abovementioned interventions may greatly improve the outcome.

In summary, this study highlighted that a positive attitude exists among the general public towards ADR reporting. Moreover, the general public was welcoming to the idea of receiving further education about this process. The study had a limitation of sampling as it gathered few responses from geriatrics and participants who were employed in the health sector. The addition of responses from those participants would have improved the representativeness of the study and might have further clarified the findings. Nonetheless, the sample size and choice of statistical techniques used to elaborate the findings from this population are strong enough to highlight the phenomenon with significance.

## 5. Conclusions

The results of this study highlighted that although the population had a high score for knowledge of medicines and tendency to report ADRs, the score for knowledge regarding medication safety was unsatisfactory. The results suggest that further education should be conducted for the population of Saudi Arabia regarding the Saudi NPC and the ADRs reporting system. The attitude of the general public towards medicine knowledge seeking and ADR reporting was positive.

## Figures and Tables

**Table 1:** Content validation.

Panelist	Items																		
	1	2	3	4	5	6	7	8	9	10	11	12	13	14	15	16	17	18	19
1.	✓	✓	✓	✓	✓	✓	✓	✓	✓	✓	✓	✓	✓	✓	✓	✓	✓	✓	✓
2.	✓	✓	✓	✓	✓	✓	✓	✓	✓	✓	✓	✓	✓	✓	✓	✓	✓	✓	✓
3.	✓	x	✓	✓	✓	✓	✓	✓	✓	✓	✓	x	✓	✓	✓	✓	✓	✓	✓
4.	✓	✓	✓	✓	✓	✓	✓	✓	✓	✓	✓	✓	✓	✓	✓	✓	✓	✓	✓
5.	✓	✓	✓	✓	✓	✓	✓	✓	✓	✓	✓	✓	✓	x	✓	✓	✓	✓	✓
6.	✓	✓	✓	✓	✓	✓	✓	✓	✓	✓	✓	✓	✓	✓	✓	✓	✓	✓	✓
7.	✓	✓	✓	✓	✓	✓	x	✓	✓	✓	✓	✓	✓	✓	✓	✓	✓	✓	✓
8.	✓	✓	✓	✓	✓	✓	✓	✓	✓	✓	✓	✓	✓	✓	✓	✓	✓	✓	x

**Table 2:** Scoring criteria of questionnaire.

Item # In Questionnaire	**Section 1: Knowledge of Medicine on a Scale of 0 to 6.** **(0 = Poor Knowledge, 6 = Good Knowledge)**	Score
4.	When you have to take a drug, do you know what it is for?	
	Yes	2
	No	0
	Sometimes	1
	I do not know	0
5.	When you have to take a drug, do you know when and with what frequency you have to take it?	
	Yes	2
	No	0
	Sometimes	1
	I do not know	0
6.	When you have to take a drug, do you know how long you have to take it for?	
	Yes	2
	No	0
	Sometimes	1
	I do not know	0
	**Section 2: Knowledge Regarding Medication Safety on a Scale of 0 to 10.** **(0 = Poor Knowledge, 10 = Good Knowledge)**	
7.	Do you read the patient information leaflet for medicines?	
	Always	2
	Sometimes	1
	Never	0
8.	Do you find the patient information leaflet difficult to understand?	
	Yes	0
	No	2
	Sometimes	1
10.	What does an adverse drug reaction (ADR) mean?	
	Any effect from the medication	0
	Unexpected reaction after taking the normal dose	2
	Expected reaction after taking the normal dose	0
	I do not know	0
11.	Do you ask or search about your medication’s ADR?	
	Always	2
	Sometimes	1
	Rarely	0.5
	Never	0
12.	Which of the following resources do you use to search or ask about ADR?	
	Asking the physician	2
	Asking the pharmacist	2
	Internet	2
	Patient Information Leaflet (PIL)	2
	I do not search about it.	0
	**Section 3: Tendency to Report a Suspected ADR on a Scale of 0 to 8.** **(0 = Lowest, 8 = Highest)**	
13.	In your opinion who should be responsible for reporting a suspected ADR from medications?	
	Health care professional	0
	Consumers	2
	All of the above	2
14.	Have you heard about Saudi National Pharmacovigilance Center (NPC)?	
	Yes	2
	No	0
16.	If you have experienced an ADR before, who did you report to from the following?	
	Health Practitioner	2
	Saudi National Pharmacovigilance Center (NPC)	2
	Did not report it	0
	Did not experience	2
17.	Do you know that consumer can directly report suspected ADR through Saudi vigilance program (NPC)?	
	Yes	2
	No	0

**Table 3:** Demographics.

Variables	Frequency (*n*)	Percent (%)
Age		
18–30	498	54.4
31–45	257	28.1
46–64	152	16.6
Older than 65	8	0.9
Gender		
Female	602	65.8
Male	313	34.2
Level of education		
Middle School	16	1.7
High school	261	28.5
Diploma	106	11.6
Bachelors	486	53.1
Higher education	46	5
Occupation		
Health sector employee	64	7
Non-health sector employee	315	34.4
Un-employed/home maker	151	16.5
Health Student	231	25.2
Non-Health Student	154	16.8
Social Status		
Single	410	44.8
Married	505	55.2

**Table 4:** Medication information.

Characteristics	Frequency (*n*)	Percent (%)
In the last three months, have you taken any drug by medical prescription?		
Yes	525	57.9
No	377	42.1
In the last three months, have you taken any drug not by medical prescription?		
Yes	483	52.8
No	416	45.5
I do not know	16	1.7
If yes, who recommended it?		
Pharmacist	187	20.4
Health practitioner	50	5.5
Friends and family	83	9.1
Apothecary	5	0.5
Social Media	12	1.3
Myself	228	24.9
When you have to take a drug, do you know what it is for?		
Yes	790	86.3
No	12	1.3
Sometimes	112	12.2
I do not know	1	0.1
When you have to take a drug, do you know when and with what frequency you have to take it?		
Yes	773	84.5
No	30	3.3
Sometimes	110	12
I do not know	2	0.2
When you have to take a drug, do you know how long you have to take it for?		
Yes	677	74
No	74	8.1
Sometimes	150	16.4
I do not know	14	1.5

**Table 5:** Medication safety.

Characteristics	Frequency (*n*)	Percent (%)
Do you read the patient information leaflet (PIL) for medicines? ^1^		
Always	394	43.1
Sometimes	454	49.6
Never	67	7.3
Do you find the patient information leaflet (PIL) difficult to understand?		
Yes	66	7.2
No	524	57.3
Sometimes	325	35.5
Which part of the patient information leaflet (PIL) do you read?		
All	484	52.9
Indication	180	19.7
Dose and instruction	112	12.2
Side effects	139	15.2
What does an ADR mean? ^2^		
Any effect from the medication	332	36.3
Unexpected reaction after taking the normal dose	340	37.2
Expected reaction after taking the normal dose	222	24.3
I do not know	21	2.3
Do you ask or search about your medication’s ADR?		
Always	185	20.2
Sometimes	372	40.7
Rarely	209	22.8
Never	149	16.3
Which of the following resource do you use to search or ask about ADR?		
Asking the physician	195	21.3
Asking the pharmacist	121	13.2
Internet	218	23.8
Patient information leaflet (PIL)	267	29.2
I did not search about it.	114	12.5

^1^ Patient information leaflet; ^2^ Adverse drug reaction.

**Table 6:** ADR reporting.

Characteristics	Frequency (*n*)	Percent (%)
In your opinion, who should be responsible for reporting possible ADR from medications?		
Healthcare professionals (HCPs)	357	39
Consumers	21	2.3
All of the above	537	58.7
Have you ever experienced an ADR?		
Yes	146	16
No	619	67.7
I do not know	150	16.4
If you have experienced an ADR before who did you report to?		
Health Practitioner	74	8.1
Saudi National Pharmacovigilance Center (SNPC)	1	0.1
Did not report it	71	7.8
Did not experience	769	84
Have you heard about Saudi National Pharmacovigilance Center (SNPC)?		
Yes	82	9
No	833	91
Do you know that consumer can directly report suspected ADR through Saudi vigilance program in NPC?		
Yes	88	9.6
No	827	90.4
Do you agree that this reporting of suspected ADR should be available for public?		
Yes	852	93.1
No	46	5
I do not know	17	1.9
In your opinion, should consumers receive more information about ADR reporting.		
Yes	828	90.5
No	9	1
I do not know	78	8.5

**Table 7:** Bivariate analysis.

Variables	Knowledge of Medicine (K1)		Knowledge about Medication Safety (K2)		Tendency to Report Suspected ADR (T1)	
	Mean (SD)	MD (95% CI)	Mean (SD)	MD (95% CI)	Mean (SD)	MD (95% CI)
**Age**						
Up to 45 years	5.43 (1.11)	−0.24 (−0.42, −0.05) **	6.02 (1.74)	0.43 (0.14, 0.73) **	3.46 (1.62)	0.12 (−0.15, 0.39) ^ns^
More than 45 years	5.66 (0.89)		5.59 (1.64)		3.34 (1.30)	
**Gender**						
Female	5.52 (1.02)	0.14 (−0.01, 0.29) ^ns^	6.14 (1.78)	0.57 (0.34, 0.79) ***	3.53 (1.67)	0.27 (0.07, 0.47) **
Male	5.37 (1.17)		5.57 (1.57)		3.26 (1.35)	
**Level of education**						
Up to high school	5.42 (1.09)	−0.07 (−0.23, 0.08) ^ns^	5.88 (1.67)	−0.09 (−0.34, 0.15) ^ns^	3.39 (1.39)	−0.06 (−0.27, 0.15) ^ns^
Higher education	5.49 (1.07)		5.97 (1.76)		3.45 (1.65)	
**Employment Status**						
Employed	5.61 (0.93)	0.24 (0.11, 0.38) ***	5.83 (1.65)	−0.19 (−0.42, 0.04) ^ns^	3.43 (1.49)	0.01 (−0.21, 0.20) ^ns^
Un-employed	5.37 (1.15)		6.02 (1.79)		3.44 (1.63)	
**Social Status**						
Single	5.19 (1.28)	−0.49 (−0.63, −0.35) ***	5.80 (1.81)	−0.25 (−0.48, −0.03) *	3.42 (1.63)	−0.03 (−0.24, 0.16) ^ns^
Married	5.68 (0.81)		6.05 (1.66)		3.45 (1.52)	

SD = Standard Deviation, MD = Mean Difference; * = *p* < 0.05, ** = *p* < 0.01, *** = *p* < 0.001, ^ns^ = Not Significant; (*n* = 915).

**Table 8:** Multivariable analysis.

Variables	Model for Knowledge of Medicine (K1) ^£^			Model for Knowledge about Medication Safety (K2) ^¥^		
	Coefficient (β) and (95% CI of β)	*p*-Value	VIF	Coefficient (β) and (95% CI of β)	*p*-Value	Variance Inflation Factor
**Age** (≤45 years vs. >45 years)	−0.003 (−0.21, 0.19)	0.928	1.23	0.011 (−0.17, 0.23)	0.756	1.20
**Gender** (Female vs. Male)	---	---	---	−0.093 (−0.37, −0.07)	0.005	1.04
**Employment Status** (Employed vs. Un-employed)	0.007 (−0.15, 0.18)	0.850	1.43	---	---	---
**Social Status** (Single vs. Married)	0.233 (0.34, 0.67)	*p* < 0.001	1.45	0.235 (0.36, 0.66)	*p* < 0.001	1.18

^£^ ANOVA (F = 16.67, *p* < 0.001), R^2^ = 0.23; ^¥^ ANOVA (F = 19.49, *p* < 0.001), R^2^ = 0.2; (*n* = 445).
